# Evaluation of Antiproliferative Properties of CoMnZn-Fe_2_O_4_ Ferrite Nanoparticles in Colorectal Cancer Cells

**DOI:** 10.3390/ph17030327

**Published:** 2024-03-01

**Authors:** Venkatesha Narayanaswamy, Bilal Rah, Imaddin A. Al-Omari, Alexander S. Kamzin, Hafsa Khurshid, Jibran Sualeh Muhammad, Ihab M. Obaidat, Bashar Issa

**Affiliations:** 1 Research Institute of Medical & Health Sciences, University of Sharjah, Sharjah P.O. Box 27272, United Arab Emirates; venkateshnrn@gmail.com (V.N.); brah@sharjah.ac.ae (B.R.); 2 Department of Physics, Sultan Qaboos University, P.O. Box 36, Muscat 123, Oman; 3 Ioffe Physical Technical Institute, 194021 St. Petersburg, Russia; 4 Department of Physics, College of Sciences, University of Sharjah, Sharjah P.O. Box 27272, United Arab Emirates; hkhurshid@sharjah.ac.ae; 5 Department of Basic Medical Sciences, College of Medicine, University of Sharjah, Sharjah P.O. Box 27272, United Arab Emirates; jmuhammad@sharjah.ac.ae; 6 Department of Medical Diagnostic Imaging, College of Health Sciences, University of Sharjah, Sharjah P.O. Box 27272, United Arab Emirates; 7 Department of Biomedical Engineering, Faculty of Engineering and Natural Sciences, Istinye University, Istanbul 34010, Turkey

**Keywords:** hyperthermia, superparamagnetic, specific absorption rate, apoptosis, antiproliferation

## Abstract

The PEG-coated ferrite nanoparticles Co_0.2_Mn_0.6_Zn_0.2_Fe_2_O_4_ (X1), Co_0.4_Mn_0.4_Zn_0.2_Fe_2_O_4_ (X2), and Co_0.6_Mn_0.2_Zn_0.2_Fe_2_O_4_ (X3) were synthesized by the coprecipitation method. The nanoparticles were characterized by XRD, Raman, VSM, XPS, and TEM. The magnetic hyperthermia efficiency (MH) was determined for PEG-coated nanoparticles using an alternating magnetic field (AMF). X2 nanoparticles displayed the highest saturation magnetization and specific absorption rate (SAR) value of 245.2 W/g for 2 mg/mL in a water medium. Based on these properties, X2 nanoparticles were further evaluated for antiproliferative activity against HCT116 cells at an AMF of 495.25 kHz frequency and 350 G strength, using MTT, colony formation, wound healing assays, and flow cytometry analysis for determining the cell viability, clonogenic property, cell migration ability, and cell death of HCT116 cells upon AMF treatment in HCT116 cells, respectively. We observed a significant inhibition of cell viability (2% for untreated control vs. 50% for AMF), colony-forming ability (530 cells/colony for untreated control vs. 220 cells/colony for AMF), abrogation of cell migration (100% wound closure for untreated control vs. 5% wound closure for AMF), and induction of apoptosis-mediated cell death (7.5% for untreated control vs. 24.7% for AMF) of HCT116 cells with respect to untreated control cells after AMF treatment. Collectively, these results demonstrated that the PEG-coated (CoMnZn-Fe_2_O_4_) mixed ferrite nanoparticles upon treatment with AMF induced a significant antiproliferative effect on HCT116 cells compared with the untreated cells, indicating the promising antiproliferative potential of the Co_0.4_Mn_0.4_Zn_0.2_Fe_2_O_4_ nanoparticles for targeting colorectal cancer cells. Additionally, these results provide appealing evidence that ferrite-based nanoparticles using MH could act as potential anticancer agents and need further evaluation in preclinical models in future studies against colorectal and other cancers.

## 1. Introduction

The ability to tune the magnetic properties of nanoparticles is essential to enhancing the efficiency of nanoparticles in biomedical applications like drug delivery, MRI contrast agents, magnetic separation of biological markers, magnetic hyperthermia, and magneto-resistive biosensing [1,2,3,4,5]. Magnetic hyperthermia uses AMF to generate local heating at the targeted sites to elevate the temperature above 42 °C for significant time results in the death of cancer cells [6]. To obtain MNPs with suitable magnetic properties for MH, the particle sizes should be close to the superparamagnetic threshold to minimize aggregation by interparticle interactions [7]. MNPs should be biocompatible and must have easy clearance away from the human body and possess low bioaccumulation. The particles should possess a high SAR value, which is the efficiency indicator for magnetic heating. Magnetic nanoparticle heating efficiency depends on magnetic anisotropy, saturation magnetization, and complex susceptibility [3]. Neel and Brownian relaxation are the key processes through which superparamagnetic nanoparticles generate magnetic heating. The Neel relaxation process of nanoparticles is mainly affected by magnetic anisotropy whereas Brownian relaxation depends mainly on the viscosity of the cellular medium [8,9]. The relaxation process is optimized by shape, composition, core-shell geometry, and functionalization of the nanoparticles. Alberto et al. have studied the effect of composition in different Co and Zn ion substituted Fe_3_O_4_ nanoparticles, obtained with the organometallic decomposition method on SAR. They reported the compositional optimization of cube-shaped particles where the magnetic properties are tuned to achieve SAR values of 884 to 386.4 W/g for magnetic nanoparticles in the size range of 30–40 nm [10]. In our recent report, we studied the effect of composition on the magnetic heating of Co_x_Mn_1-x_Fe_2_O_4_ (*x* = 0, 0.2, 0.4, 0.6, 0.8, and 1.0) nanoparticles synthesized with the coprecipitation method. The composition with x = 0.2 (Co_0.2_Mn_0.8_Fe_2_O_4_) and saturation magnetization of 57.41 emu/g showed an SAR value of 190.61 W/g at an AMF frequency of 765.95 kHz and 350 G strength [11]. Mixed ferrite nanoparticles have shown high SAR values for Zn-doped CoFe_2_O_4_ nanoparticles, indicating the potential of distribution of divalent metal ions in a spinel lattice with magnetic heating [12]. Magnetic hyperthermia kills cancer cells through several pathways. Heat generated from the MNPs induces apoptosis (programmed cell death) in cancer cells [13]. The heat can disrupt the tumor vasculature and reduce blood flow, leading to tumor cell death. The heat can cause structural changes in proteins and DNA within the cancer cells, leading to their destruction. Several studies have demonstrated the ability of hyperthermia to induce apoptosis in cancer cells [13,14]. One of the advantages of magnetic hyperthermia-induced apoptosis is its selectivity for cancer cells. Tumor cells possess dense vascular networks, impeding heat dissipation and regulation of temperature. Consequently, hyperthermia induces apoptosis in these cells directly, while healthy tissue remains unaffected at its usual temperature. The heightened oxidative stress resulting from escalated metabolism further contributes to cellular demise. Tumor cells subjected to temperatures exceeding 42 °C for 30–40 min experience shortened survival times, ultimately leading to their death [15,16]. This selectivity potentially reduces the side effects associated with treatments such as chemotherapy and radiation therapy [17]. The proliferation ability of cancer cells subjected to any treatment should be analyzed for their cancer progression ability. The ability of the surviving cells to progress further can be examined by using several cellular assays, like colony formation and wound healing assays [18]. A colony-forming assay is an in vitro quantitative technique to examine the capability of a single cell to grow into a large colony [19]. Clonogenic activity is a sensitive indicator of undifferentiated cancer stem cells. In this work, we evaluated the effect of divalent metal ion (Co^2+^, Zn^2+^, and Mn^2+^) occupancy in tetrahedral and octahedral sites for tuning the magnetic properties to achieve high heating efficiency. The distribution of metal ions in a spinel lattice was examined using Raman spectroscopy on the particles synthesized using a simple coprecipitation method. We examined the effect of magnetic heating using one set of efficient nanoparticles for cell death through apoptosis and necroptosis. The MH-surviving cancer cells’ ability to proliferate was further examined by performing colony formation and wound healing assays.

## 2. Results and Discussion

Three sets of ferrite nanoparticles were synthesized by the coprecipitation method using Co, Mn, Zn, and Fe salt precursors in a water medium at pH 12–13. Mixed ferrite compositions are considered to tune the magnetic properties to achieve high heating efficiency. The mixed ferrite nanoparticles in which the divalent metal ion (Fe^2+^) was replaced by Co^2+^, Mn^2+^, Ni^2+^, and Zn^2+^ in a spinel lattice tuned the magnetic anisotropy of the nanoparticles. Magnetic anisotropy is one of the important parameters which affect the magnetic hyperthermia efficiency of the nanoparticles, causing a change in the Neel relaxation process of the superparamagnetic nanoparticles [11]. The nanoparticles synthesized were coated with PEG and characterized using XRD, Raman, XPS, and VSM. The hydrodynamic sizes of the PEG-coated nanoparticles were obtained using DLS and subsequently used for the magnetic hyperthermia treatment of cancer cells.

### 2.1. XRD

**Figure 1:**
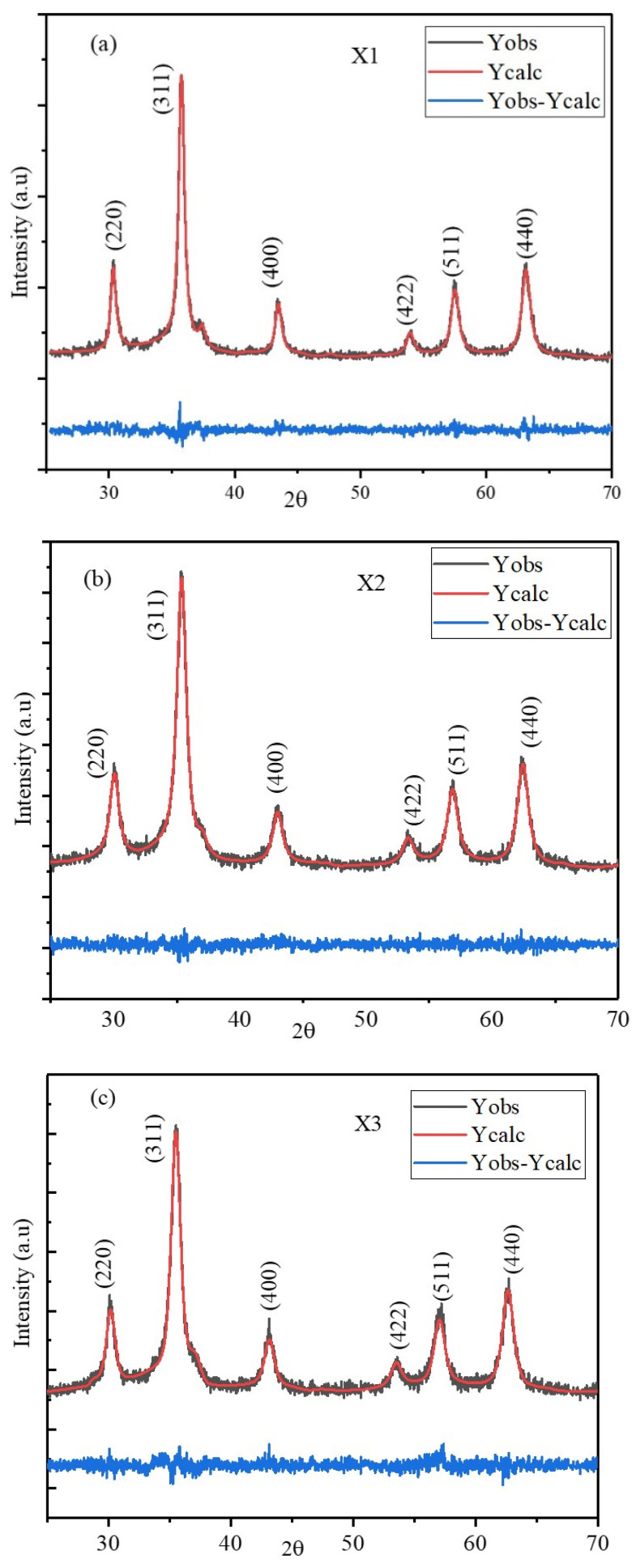
Rietveld Refinement of the XRD patterns of the (**a**) X1, (**b**) X2, and (**c**) X3 nanoparticles. (Black—experimental data, red—theoretical diffractogram, and blue—difference between the experimental and theoretical data).

The XRD patterns for the X1, X2, and X3 nanoparticles synthesized with the coprecipitation method are shown in Figure 1a,b,c. The diffraction peaks were indexed for the ferrite phase without any additional peaks corresponding to the impurity phases of ZnO, MnO_2_, Fe_2_O_3_, and CoO, which were expected from the precursors used for the synthesis [20,21]. The average crystallite sizes of the nanoparticles were obtained from FWHM of the highest intensity peak (311) using the Scherrer formula [22]. The unit cell parameters of the nanoparticles were determined through Rietveld Refinement, with their diffractogram indicating cubic symmetry (space group F3dm). Rietveld Refinement was conducted using fullprof software. Table 1 provides a summary of the refined parameters, the obtained lattice parameter, and the average sizes calculated using the Scherrer method. X1 nanoparticles having high Mn^2+^ concentrations have large crystallite sizes compared with the X2 and X3 nanoparticles. The average sizes obtained for the three sets of nanoparticles have a strong dependency on the composition of precursors used in the synthesis. The supersaturation and diffusion of the reactants determine the rate of nanoparticle nucleation and growth. The pH and ionic strength of the reaction mixture regulate how easily the reactants diffuse to the growth site. The composition-dependent shift in XRD peak positions is shown in Figure 1a,b,c. The peaks for the particles having high Mn^2+^ concentrations (X1) were shifted to the lower diffraction angle due to the larger lattice size. The lattice constants of the mixed ferrite nanoparticles are listed in the Supplementary Data in Table S1. The lattice constants of pure MnFe_2_O_4_ (8.4889 Å), CoFe_2_O_4_ (8.3891 Å), and ZnFe_2_O_4_ (8.3970 Å) were reported for particles synthesized by a similar method [11,23]. A decrease in the lattice constant for the mixed nanoparticles with an increase in the Co^2+^ concentration was expected, as the ionic radii of Co^2+^ were smaller than those of Mn^2+^.

### 2.2. TEM Image of X2 Nanoparticles

**Figure 2:**
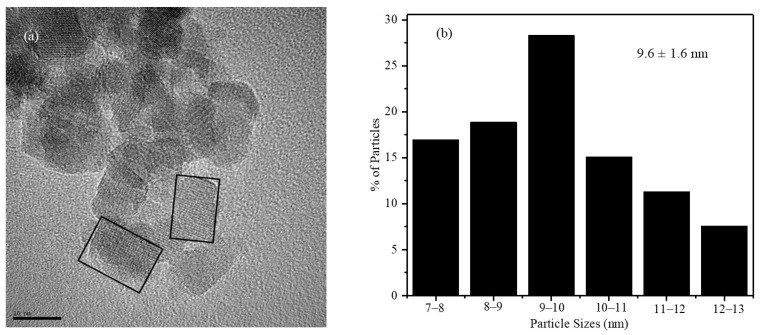
(**a**) TEM bright-field image (solid lines are the indication of the shape of the nanoparticles) and (**b**) size distribution histograms of the X2 nanoparticles.

A TEM bright-field image and the size distribution plot obtained for the X2 nanoparticles are shown in Figure 2a,b, respectively. Bright-field TEM images show that the X2 nanoparticles synthesized have a rectangular geometry, which is marked in the TEM image as solid lines. The nanoparticles possessed significant size distribution, as shown in the TEM image and size distribution histogram. The size distribution shown in Figure 2b was obtained by measuring the sizes of more than 200 individual particles from different TEM images. The average size of the nanoparticles was calculated from the histograms as 9.6 ± 1.6 nm. The average crystallite sizes of the nanoparticles obtained from the Scherrer method and particle sizes with error bars obtained from the TEM images are comparable. The particle sizes obtained from the TEM images were expected to be greater than the crystallite size. The particle size distributions were carried out by counting the size of roughly 200 particles, whereas the XRD sizes (10.2 nm) were obtained from a large amount of the sample. From the size distribution histogram, it is clear that the sizes of the particles vary from 7 to 13 nm, indicating large size distributions. The particle sizes calculated with error bars (9.6 ± 1.6 nm) obtained from the TEM images are in good agreement with the average crystalline sizes calculated from XRD.

### 2.3. Raman Spectra of X1, X2, and X3 Nanoparticles

**Figure 3:**
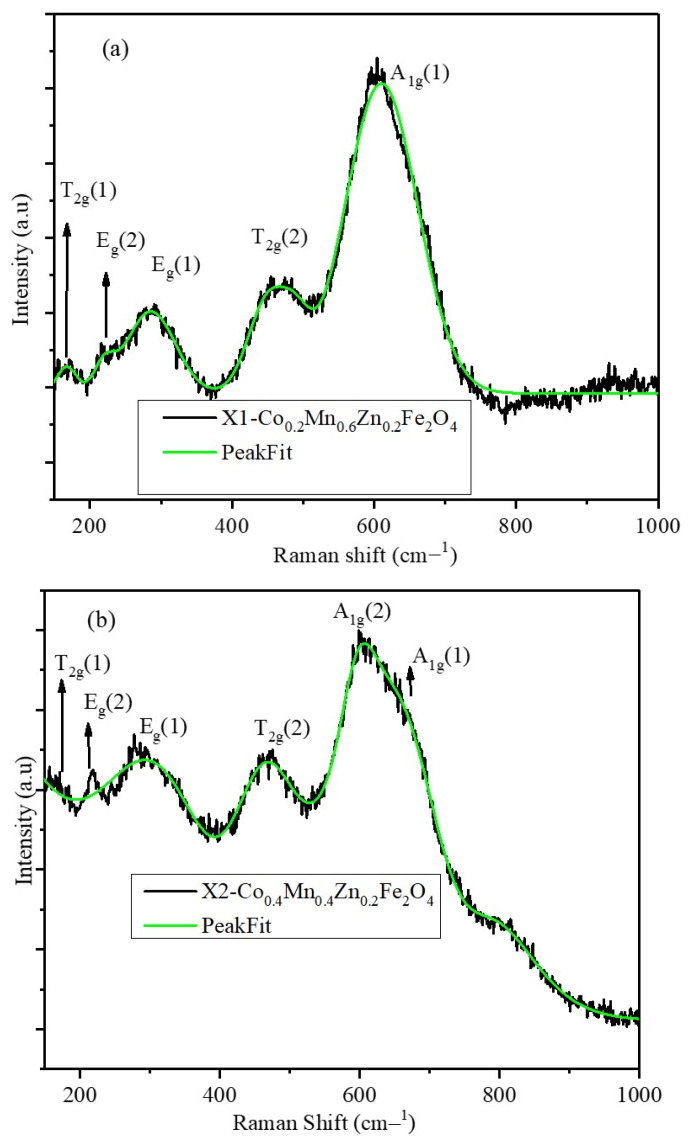

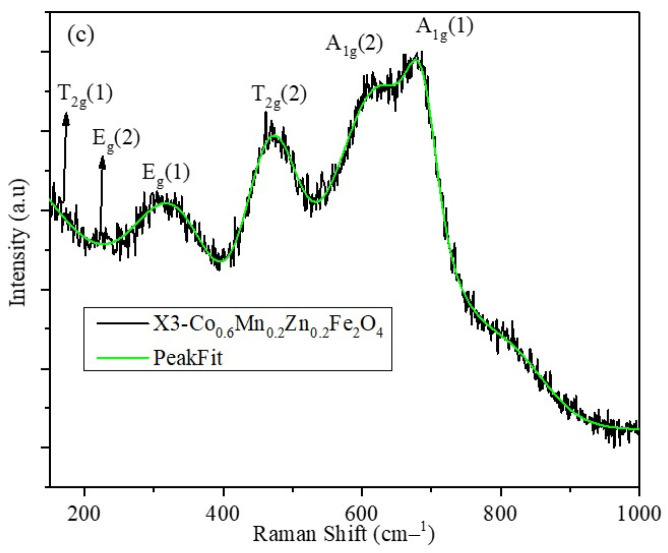
Raman spectra of X1, X2, and X3 nanoparticles (the green line indicates the peak fitting).

Raman spectra of the X1, X2, and X3 nanoparticles are shown in Figure 3a–c. The peak fitting is shown by the solid green-colored lines. The peak position and intensity of the Raman spectra depend on the distribution of metal ions among the tetrahedral and octahedral sites of the spinel lattice. In a normal spinel (MnFe_2_O_4_), divalent Mn^2+^ ions occupy tetrahedral sites, while octahedral sites are occupied by trivalent Fe^3+^ whereas in the case of inverse spinel (CoFe_2_O_4_), divalent Co^2+^ ions occupy half of the octahedral sites and trivalent Fe^3+^ ions are distributed equally among tetrahedral and octahedral sites [24,25]. The Raman vibrational modes obtained from the group theory calculations consist of A_1g_ + E_g_+ three T_2g_ phonon bands. The peak positions and intensity ratios are listed in Table 2. A1g(1) and A1g(2) correspond to the symmetric stretching of oxygen atoms with respect to Fe and Co ions (Fe-O and Co-O bonds in tetrahedral sites). The intensity ratios of A1g(1) and A1g(2) peaks provide information about the degree of the inverse nature of the spinel structure [26]. The X1 nanoparticles displayed a normal spinel structure, which is indicated by a single A1g peak at 605.4 cm^−1^ which is similar to the peak positions reported for the MnFe_2_O_4_ nanoparticles [11], whereas the X2 and X3 nanoparticles have a split in the A_1g_ peak, which is due to the redistribution of the Co^2+^ and Fe^3+^ ions. The intensity ratio of the A_1g_(1) and A_1g_(2) peaks indicates that the X3 nanoparticles have a complete inverse spinel structure and the X2 nanoparticles possess a mixed spinel structure. The observed site occupancy of the divalent and trivalent cations has a significant effect on the magnetic properties and magnetic hyperthermia of the nanoparticles.

### 2.4. DLS, FTIR, and XPS of X1, X2, and X3 Nanoparticles

The nanoparticles synthesized were further characterized using DLS, FTIR, and XPS; the details are provided in the Supplementary Data. The hydrodynamic sizes of the X1, X2, and X3 nanoparticles are shown in Figure S1a (Supplementary Data). The hydrodynamic sizes vary in the range of 100–200 nm. The zeta potentials of the synthesized and PEG-coated nanoparticles are shown in Figure S1b–d. The zeta potential of the PEG-coated nanoparticles shifted to lower values of −40 to −60 mV and the synthesized nanoparticles have a zeta potential value of −30 to −38 mV. FTIR analysis was used to identify PEG in functionalized ferrite nanoparticles. The FTIR spectra of synthesized nanoparticles and PEG-coated nanoparticles are shown in Figure S2a–c (Supplementary Data). In the FTIR spectra of PEG-coated ferrite nanoparticles, additional peaks corresponding to bending vibrations of -C-O-C- (1100–1250 cm^−1^), out-of-plane bending vibrations of -CH (833 cm^−1^), and bending vibrations of -CH_2_ (1385 cm^−1^) are observed. The XPS peak positions of the 2p_3/2_ and 2p_1/2_ peaks corresponding to the cations are listed in Table S1 (Supplementary Data). The Fe^3+^ showed a major peak at 711.0 eV, attributed to the peak from the Fe2p_3/2_ core-level electrons of the Fe^3+^. The intensities of the peaks indicate that the mass percentage of Fe^3+^ is identical in the three sets of nanoparticles. The peaks corresponding to the Mn^2+^ and Co^2+^ shown in Figure S3b,c (Supplementary Data) show concentration-dependent intensity; in particular, the satellite peak Co^2+^ is more significant in the spectra of X3 nanoparticles due to the higher mass percentage of Co. The Zn^2+^ peaks have identical intensity and a slight concentration-dependent shift in the peaks, in which 2p_3/2_ and 2p_1/2_ peaks shift to higher binding energy with the increase in the Co^2+^ atomic percentage.

### 2.5. Magnetic Characterization of Nanoparticles

**Figure 4:**
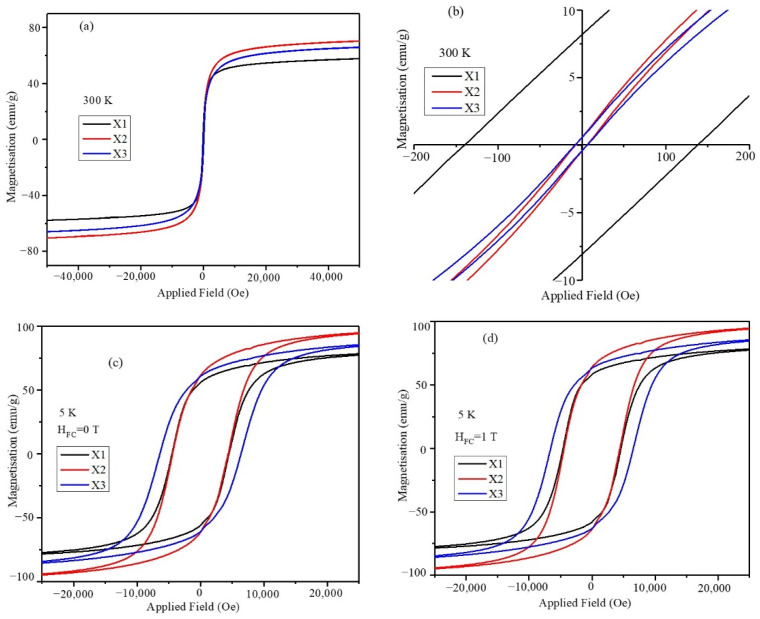
(**a**) Magnetic hysteresis loops obtained at room temperature in the field range of −5.0 T to +5.0 T for X1, X2, and X3 nanoparticles. (**b**) Zoomed portion of MH plots at the origin, (**c**) MH plots obtained at 5 K, and (**d**) MH plots of obtained at 5 K under 1 T field cooling.

Magnetic hysteresis (MH) loops obtained at room temperature and 5 K under zero field cooling and at 1 T for the mixed ferrite nanoparticles are shown in Figure 4a–d. The magnetic hysteresis loops were obtained by swapping DC magnetic fields in the range of −5.0 T to +5.0 T. The saturation magnetization (M_S_), remnant magnetization (M_R_), and coercive field (H_C_) of X1, X2, and X3 nanoparticles are listed in Table 3. The saturation magnetization of the nanoparticles is composition-dependent, and X2 has the highest among the three mixed ferrites. The particles do not show a composition-dependent trend, whereas some of the mixed Co_1-x_Zn_x_Fe_2_O_4_ ferrite particles with two divalent (Co^2+^ and Zn^2+^) metal ions show a decreasing saturation magnetization trend with increasing Zn^2+^ concentration [27]. The three sets of nanoparticles presented in this study have a fixed percentage of Zn^2+^ ions, whereas the Co^2+^ and Mn^2+^ divalent compositions are varied. Ferrites with compositions of Co_1-x_Mn_x_Fe_2_O_4_ nanoparticles show a deviation in the Ms trend compared with Zn-doped CoFe_2_O_4_. In Co_1-x_Mn_x_Fe_2_O_4_, the composition with x = 0.8 has the highest saturation magnetization compared with CoFe_2_O_4_ nanoparticles, indicating the effect of Mn^2+^ in the CoMnZn-Fe_2_O_4_ mixed ferrite nanoparticles. The room-temperature coercive field (139.8 Oe) and remnant magnetization (8.12 emu/g) of the X1 nanoparticles are significantly high compared with those for the X2 and X3 particles, which is attributed to the larger sizes (15 nm) of the X1 nanoparticles and composition. The X2 and X3 nanoparticles have negligible M_R_ (0.5 emu/g) and H_C_ (7 Oe) at room temperature, whereas the saturation magnetization (M_S_) is high compared with the X1 nanoparticles. The magnetic properties of the three sets of nanoparticles were compared at 5 K. Interestingly, the magnetic properties at low temperature do not follow the trends shown at room temperature. The nanoparticles at low temperature (5 K) have a high remnant magnetization and coercive field; the X3 particles show a high H_C_ (6382.3 Oe) value whereas the X1 and X2 nanoparticles have similar H_C_ values of 4400 Oe. The remnant magnetization of the X2 nanoparticles is 66.62 emu/g. Effective magnetic anisotropy and saturation magnetization of the nanoparticles were determined using the law of approach to saturation method with a high magnetic field [28]. The fitting was calculated in the field range in which all the magnetic spins are oriented in the direction of the magnetic field. The magnetic properties obtained from the MH plots for the three sets of nanoparticles are shown in Table 3.

**Figure 5:**
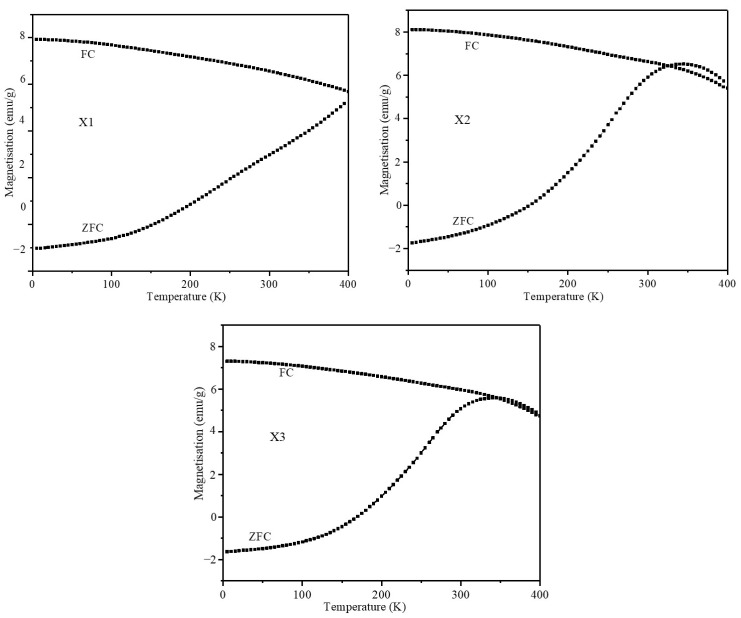
ZFC and FC plots obtained under 100 Oe field for X1, X2, and X3 nanoparticles.

Temperature-dependent magnetization plots of the nanoparticles were obtained under ZFC- and FC-cooled conditions. The nanoparticles were cooled to 2 K in a zero magnetic field before being exposed to 100 Oe to record the ZFC plots. To obtain the ZFC magnetization curves, the sample was heated from 2 to 400 K and the magnetization was measured. Then, the temperature decreased to 2 K while magnetization was recorded, giving the FC curve. The ZFC and FC plots of the nanoparticles are shown in Figure 5. ZCF and FC plots of the X1 nanoparticles indicate that the nanoparticles do not possess a well-defined blocking temperature due to their multidomain nature or large size distribution, and their observed coercivity and remanent magnetization are in correlation. Though the blocking temperature of the X2 (345.0 K) and X3 (340.0 K) nanoparticles is higher than the room temperature, the coercivity and remnant magnetization of the nanoparticles are negligible. These properties are essential for the nanoparticles to be used in magnetic hyperthermia for in vitro studies to prevent the agglomeration of particles. The nanoparticles showed high stability in cellular media without any aggregation during AMF treatment for a significant time.

### 2.6. Magnetic Hyperthermia of Co,Mn,Zn-Fe_2_O_4_ Nanoparticles in Water and Agar Ferrogel

**Figure 6:**
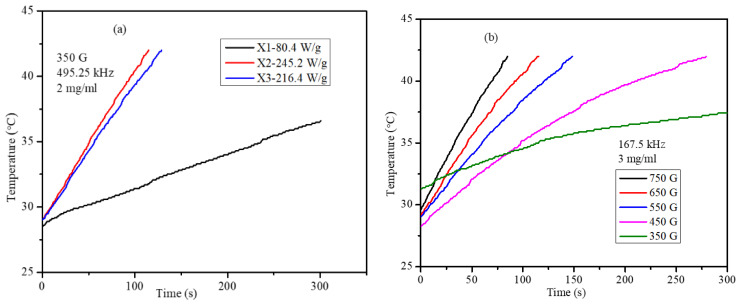

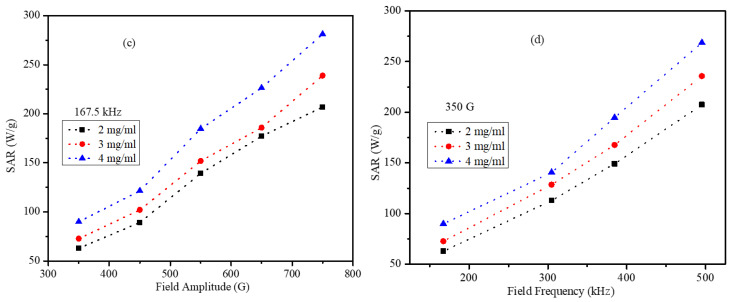
(**a**) Heating profiles of PEG-coated X1, X2, and X3 nanoparticles at AMF 495.25 kHz and 350 G. (**b**) Field-dependent heating profiles of X2 nanoparticles of 3 mg/mL agar ferrogel at 167.5 kHz. (**c**) Field amplitude-dependent SAR values of X2 nanoparticles at 167.5 kHz. (**d**) Frequency-dependent SAR values of the X2 nanoparticles at 350 G field amplitude.

Magnetic hyperthermia of the PEG-coated nanoparticles in water media and agar ferrogel was obtained under AMF using field frequencies of 495.25, 384.5, 304.75, and 167.5 kHz and field strength of 350–750 G. The heating profiles and SAR values are provided in Figure 6a for X1, X2, and X3 nanoparticles in a water medium under AMF with a frequency of 495.25 kHz and field strength of 350 G. These heating profiles show that X2 nanoparticles have the highest heating efficiency, with an SAR value of 245.2 W/g. The high SAR value of X2 (Co_0.4_Mn_0.4_Zn_0.2_Fe_2_O_4_) nanoparticles is attributed mainly to their high saturation magnetization, *M*_s_, compared with those for X1 and X3. According to the linear response theory (LRT), SAR depends on the square of *M*_s_ [29]. The heating ability of the superparamagnetic nanoparticles mainly depends on factors like saturation magnetization, magnetic anisotropy, and particle size. From the magnetic properties listed in Table 3, it is evident that the X2 and X3 nanoparticles possess higher Ms and anisotropy values compared with X1. Though the crystallite sizes of the X2 and X3 nanoparticles are comparable, optimum site occupancy of metal ions in the tetrahedral and octahedral sites is required to achieve high heating efficiency. Since X2 nanoparticles showed high heating efficiency, further SAR and in vitro studies were conducted using PEG-coated X2 nanoparticles. The concentration-dependent SAR values were obtained at a constant AMF frequency and constant field strength using 2 and 3 mg/mL X2 agar ferrogel nanoparticles. Field strength-dependent SAR (at 167.5 Hz) values of the X2 nanoparticles at particle concentrations of 2 and 3 mg/mL are shown in Figure 6c; the SAR values show nearly linear dependency on the field amplitude. Field frequency-dependent SAR (at 350 G) values of the X2 nanoparticles at particle concentrations of 2 and 3 mg/mL are shown in Figure 6d; the SAR values show nearly linear dependency on the field frequency. The concentration of the nanoparticles has a mild effect on the heating ability, which is indicated in Figure 6c,d, which show that 3 mg/mL has slightly higher values compared with the 2 mg/mL agar ferrogel. The slight deviation from the expectations of the LRT could be attributed to the size distribution of the particles.

### 2.7. MTT Cytotoxicity Assay

**Figure 7:**
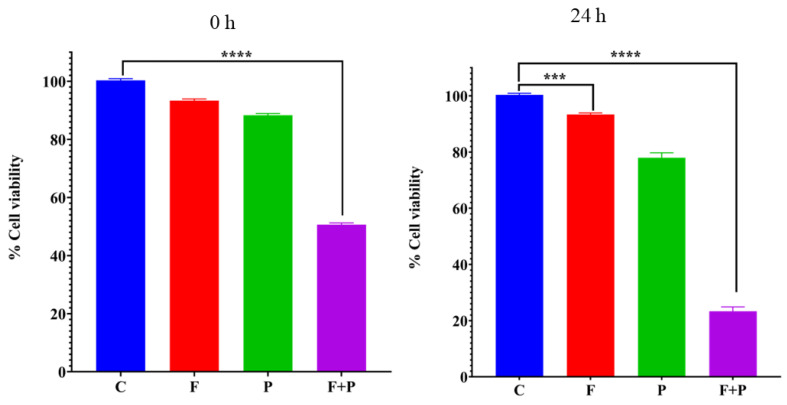
MTT assay of HCT116 cells treated with AMF after 0 and 24 h of treatment (**** and *** indicates that *p* value is less than 0.0001 and 0.001 respectively).

Magnetic hyperthermia for evaluation of antiproliferative activity in HCT116 cells was observed by treating cells with 1 mg/mL of PEG-coated X2 nanoparticles. The cells that were treated with AMF 495.25 kHz and 350 for 30 min reached a saturation temperature of 47 °C from the magnetic heating. The intrinsic toxicity of the magnetic field and nanoparticles was obtained by treating HCT116 cells in similar conditions to those used for AMF treatment. The cellular viability was determined using an MTT assay at 0 and 24 h time points after AMF treatment. HCT116 cells were grouped in four sets to determine magnetic cytotoxicity; they are labeled as follows: control cells (C), cells treated with magnetic field (F), cells treated with particles (P), and cells treated with particles and AMF (P + F). The cell viability of the HCT116 cells treated with particles and AMF that were obtained with respect to control cells after 0 h and 24 h treatments are shown in Figure 7. The viability of HCT116 cells decreased to 50% immediately (0 h) after treatment and further decreased to 25% after 24 h of treatment. The results for the cell proliferation assay for cells treated with AMF showed a significant difference with respect to the untreated control group at *p* < 0.0001. The viability of cancer cells treated with the field alone was similar to that of the control at different time points, indicating a lack of any toxicity from the magnetic field. Cells treated with particles alone showed slight toxicity only after 24 h, which could be due to the mechanical pressure of particles cultured on top of the cells in the culture dishes.

### 2.8. Apoptosis Assay

**Figure 8:**
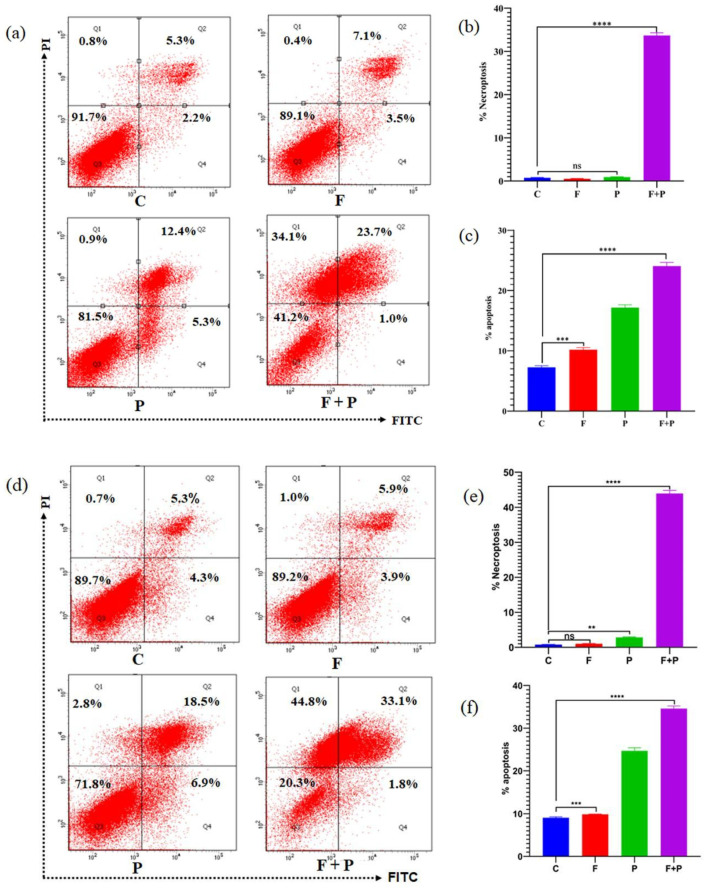
Apoptosis assay of HCT116 cells treated with AMF after 0 h: (**a**) flow cytometry assay, (**b**) percentage of necroptosis, and (**c**) percentage of total apoptosis. Apoptosis assay of HCT116 cells treated with AMF after 24 h: (**d**) flow cytometry assay, (**e**) percentage of necroptosis, and (**f**) percentage of total apoptosis (****, ***, and ** indicates that *p* value is less than 0.0001, 0.001, and 0.01 respectively).

Annexin V-FITC apoptosis staining was used to detect cell death by staining phosphatidylserine molecules which had translocated to the outside of the cell membrane after AMF treatment and nanoparticles using flow cytometry. Flow cytometry analysis of the HCT116 cells treated with AMF, subsequently stained after 0 h of the treatment with propidium iodide (PI) and FITC, are shown in Figure 8a. Treatment of cancer cells above 42 °C using MNPs caused structural changes in proteins and DNA within the cancer cells, leading to their cell death [30]. This cell death can occur through several mechanisms, including oxidative stress, which leads to membrane damage, and disruption of the cellular metabolic pathways. The cell deaths due to AMF treatment are classified based on the mapping from the flow cytometry analysis as necroptosis, early apoptosis, and late apoptosis. Necroptosis is a form of programmed cell death that occurs in response to various types of cellular stress, such as viral infection or oxidative stress or even mechanical stress [31]. Unlike apoptosis, which is a well-characterized form of programmed cell death that involves controlled dismantling of cells, necroptosis results in the rupture of cells and the release of cellular contents into the extracellular environment, triggering an inflammatory response. Necroptosis is initiated by a signaling pathway involving receptor-interacting protein kinases (RIPKs), particularly RIPK1 and RIPK3, which activate a protein called mixed lineage kinase domain-like protein (MLKL). MLKL is then translocated to the plasma membrane and forms pores that disrupt the integrity of the membrane, leading to cell death [32]. To investigate whether AMF induces a cell death mechanism in HCT116 cells, we performed flow cytometry. Upon AMF treatment of HCT116 cells at 0 h with respect to the untreated control, our flow cytometry results revealed a significant percentage of the cell population was undergoing apoptosis, as shown in Figure 8c; intriguingly, we also observed a significant population of necrotic cells in AMF treatment, as shown in Figure 8c, which might be due to mechanical stress or induction of oxidative stress in cells induced by the magnetic field. However, AMF treatment of HCT116 cells for 24 h showed significant increases in both apoptotic and necrotic cell death in HCT116 cells, as shown in Figure 8d–f. The percentage of cell death obtained from flow cytometry is comparable to the variability in cells obtained from the MTT assay. AMF-treated cells showed 34.1 and 44.8% necroptosis after 0 and 24 h treatment, which shows significant death of cancer cells.

### 2.9. Colony Formation Assay of HCT116 Cells Treated with AMF

**Figure 9:**
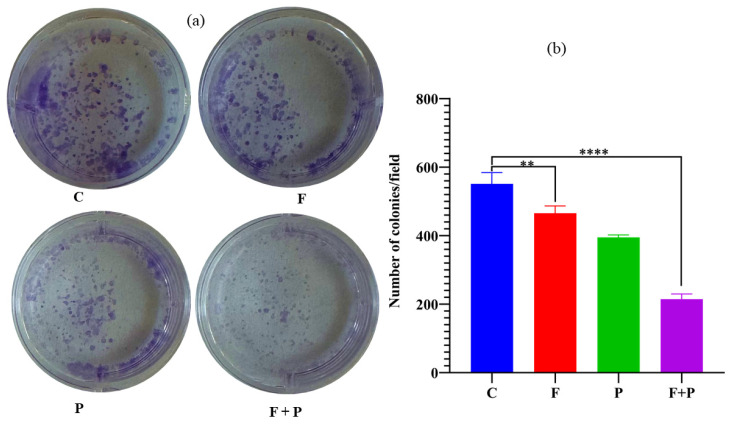
(**a**) Colonies of HCT cells treated with AMF and control cells. (**b**) Number of colonies formed under different conditions within the 6-well field (**** and ** indicates that *p* value is less than 0.0001 and 0.01 respectively).

Colony formation assays are commonly used to assess the ability of cancer cells to form colonies, which is an important characteristic of their ability to proliferate and potentially cause metastasis [33]. The effect of magnetic hyperthermia on colony formation in cancer cells was investigated by seeding the HCT116 cells which survived AMF treatment, which are shown in Figure 9a along with the C, F, and P control cells. The numbers of colonies formed in 7 days for the AMF-treated cells and control cells are shown in Figure 9b. The colony formation ability of cancer cells refers to their ability to grow and divide to form colonies in culture, which is an important hallmark of cancer. Cancer cells can grow uncontrollably, and this ability to form colonies is one of the key characteristics that allows them to invade and spread throughout the body. Untreated HCT116 cells showed a high ability to form colonies compared with the field- and particle-treated cells even though these conditions did not cause any significant toxicity in the MTT and apoptosis assays, whereas in the AMF- and particle-treated cells, the cells lost the capability to form colonies, which indicates the effect of hyperthermia treatment.

### 2.10. Wound Healing Assay of HCT116 Cells Treated with AMF

**Figure 10:**
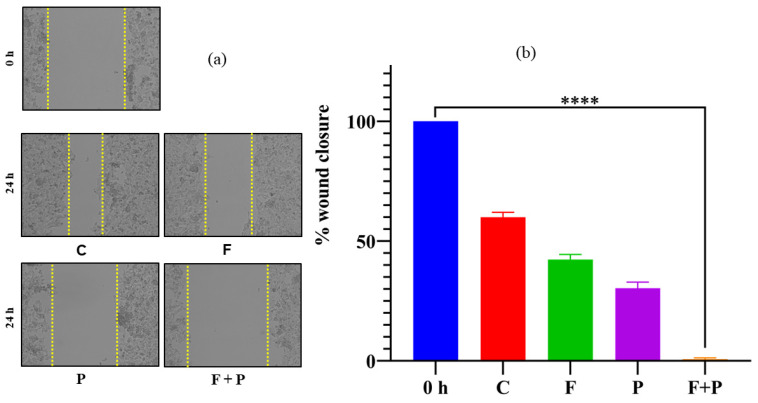
(**a**) Images of the wound created by scratch for C, F, P, and P + F cells. (**b**) Percentage of wound closure for the cells treated with AMF (**** indicates that *p* value is less than 0.0001).

The wound healing ability of cancer cells refers to their ability to migrate and proliferate to repair damaged tissue, which is an important marker of cancer progression. In normal wound healing, cells migrate to the site of injury, proliferate to replace damaged tissue, and then stop growing once the tissue is repaired [34]. However, cancer cells may continue to grow and divide uncontrollably, leading to the formation of tumors. In some cases, cancer cells may also have enhanced wound healing ability compared with normal cells. This can occur because cancer cells could have secreted factors that promote angiogenesis, or the growth of new blood vessels, which is necessary for the repair of damaged tissue. Additionally, cancer cells may also be able to invade surrounding tissue and migrate to distant sites in the body, which are key steps in the progression of metastases. The wound healing images captured at 0 h and 24 h after creating the wound are shown in Figure 10a for AMF-treated HCT116 cells along with the untreated control cells. As shown in Figure 10a,b, we observed that the hyperthermia-treated cells maintained the same wound gap as the 0 h wound, indicating that hyperthermia treatment inhibited the cell invasion and migration ability of HCT116 cells and indicating that AMF treatment exhibits potential anti-cell-migration ability. The cells treated with the magnetic field and particles alone also showed a slight inability to migrate and heal compared with the untreated cells. The percentage of wound closure of the scratch was calculated by comparing the width with the zero-hour width, as shown in Figure 10b.

Together, these results suggest that in vitro treatment of the HCT116 cells with PEG-coated X2 nanoparticles at 495.25 kHz and 350 G frequency and field strength for 25 min shows a significant antiproliferative effect using various functional assays such as MTT assays, wound healing assays, colony formation assays, and flow cytometry at 0 and 24 h after treatment with the AMF. Our results suggest that AMF exhibits significant inhibition of cell viability in HCT116 cells. Furthermore, wound healing and colony formation assays revealed that AMF treatment exhibits anti-migration and anti-clonogenic effects against the HCT116 cellular model of colorectal cancer. Additionally, flow cytometry analysis demonstrated that AMF-mediated cell death in HCT116 cells is due to apoptosis and necroptosis, indicating that AMF nanoparticles exhibit promising antiproliferative activity against the cellular model of colorectal cancer (HCT116) and the underlying cell death mechanism needs further validation in different cancer cell models and for preclinical testing.

## 3. Materials and Methods

### 3.1. Synthesis of Co, Mn, and Zn-Fe_2_O_4_ Nanoparticles

Three sets of ferrite nanoparticles, Co_0.2_Mn_0.6_Zn_0.2_Fe_2_O_4_(X1), Co_0.4_Mn_0.4_Zn_0.2_Fe_2_O_4_ (X2), and Co_0.6_Mn_0.2_Zn_0.2_Fe_2_O_4_ (X3), were synthesized using the one-step coprecipitation method in a water medium. Batch sizes of 2.5 g of each nanoparticle composition were synthesized by dissolving the MnCl_2_, CoCl_2_.6H_2_O, ZnCl_2_, and FeCl_3_ salts as listed in Table 4 in 200 mL of water and preheating them to 80 ˚C under stirring. The pH of the solution mixture was adjusted in the range of 12–13 by adding NaOH solution dropwise under constant stirring; the reaction mixture was subsequently heated at 85 °C for 1 h. The nanoparticles synthesized were cooled to room temperature and washed using a centrifuge at 8000 RPM. The nanoparticles were dried using an IR lamp and structural characterization was carried out. The nanoparticles were coated with PEG (polyethylene glycol) to make them water-dispersible and biocompatible. Amounts of 200 mg of X1, X2, and X3 nanoparticles were dispersed in 50 mL of distilled water by probe sonication for 30 min. To this dispersion, 10 mL of PEG solution of a concentration of 2.5 mg/mL was added and sonicated for 10 min and subsequently kept for 24 h. After 24 h, the nanoparticle dispersion was centrifugated at 8000 rpm to remove uncoated PEG molecules, and surfactant-coated ferrite nanoparticles were redispersed in 10 mL of water and used for hyperthermia treatment of cells.

### 3.2. Characterization of the Nanoparticles

The structural phases of the nanoparticles and the crystallites’ sizes were determined from the X-ray diffraction profile using a D8 ADVANCE Bruker XRD diffractometer with Cu-Kα radiation and a wavelength of 1.542 Å. The dc magnetic measurements were carried out using a vibrating sample magnetometer (VSM) in the Physical Properties Measurement System (PPMS) from Quantum Design. Raman spectra were obtained from the nanoparticle pellets using a NOST Raman spectrometer consisting of a diode-pumped solid-state laser operating at 532 nm with a charge-coupled detector. The TEM images of the nanoparticles synthesized with the coprecipitation method were obtained from the carbon-coated copper grid prepared by the drop drying method. Bright-field images were acquired using a Titan Themis 300 kV transmission electron microscope (TEM).

### 3.3. Magneto-Thermal Measurements

The magnetic heating efficiency of the PEG-coated nanoparticles in a water medium and agar ferrogel was obtained using a nanoScale Bio magnet hyperthermia instrument. The AMF field frequencies were 495.25, 384.50, 304.75, and 167.30 kHz and the field amplitude used was in the range of 350–750 G. Changes in the temperature of the nano dispersion under AMF with respect to time were measured using a fiber optic temperature reader. The SAR values are given by Equation (1) [35]:

(1) $$SAR(W/g)=\frac{C}{m}\frac{dT}{dt}\;$$

where C is the specific heat capacity of water (4186 JL^−1^K^−1^ at room temperature), m is the mass concentration of the magnetic material (g/L), and dT/dt is the initial slope of the temperature versus time curve. This choice was considered because at the initial stage of heating, the heat transfer between the sample and the environment is negligible and thus adiabatic conditions could be valid. In addition, temperature variations within the sample are expected to be very small in the initial heating process, and thus can be ignored.

### 3.4. MTT Assay

The viability of AMF-treated cells was determined by measuring the absorbance of the MTT [3-(4, 5-dimethylthiazol-2-yl)-2, 5-diphenyltetrazolium bromide] at 570 nm using an ELISA plate reader, the SynergyTM HTX microplate reader (BioTek, Winooski, VT, USA). Briefly, 1 × 10^4^ cells/well of AMF-treated HCT116 cells were seeded in a 96-well cell culture plate (Corning, Sigma-Aldrich Co., St. Louis, MO, USA). Control group (C) cells were maintained at 37 °C in the incubator throughout the experiment. One set of cells (F) was treated with the magnetic field without nanoparticles to examine the effect of the magnetic field. One more group of cells (P) was treated with nanoparticles without the field to study their effect alone. For the AMF treatment, cells (P + F) were treated with 1 mg/mL of X2 nanoparticles under AMF at 495.25 kHz frequency and 350 G strength. Cell viability was measured at 0 and 24 h post-AMF treatment. At the specified time intervals (0 and 24 h), the cells were washed three times with phosphate-buffered saline (PBS) to remove the nanoparticles from the cell surface, followed by the addition of 100 µL of culture medium and 10 µL of MTT solution (5 mg/mL in PBS), and incubated for 2 h under humidified air and 5% CO_2_ at 37 °C. The MTT-containing medium was then removed and formazan crystals were dissolved in 100 µL of dimethyl sulfoxide. The absorbance at 570 nm of a pink-colored solution of DMSO and dissolved formazan crystals was used to determine the cell viability percentage with respect to the control group of cells.

### 3.5. Apoptosis Assay Using Flow Cytometry

The quantification of apoptotic and necrotic cell death was studied after treatment with AMF and was determined with an apoptosis kit as per manufacturer protocol. Analysis of cell viability and the presence of apoptotic markers was conducted to determine the impact of AMF treatment on cancer cells. Briefly, 0.5 × 10^4^ cells/well in a 6-well plate were seeded. Similar to the MTT assay, four groups of cells (C) were treated with field (F), nanoparticles (P), and particles + field (P + F) along with an untreated control of HCT116 cells for 0 and 24 h in 5% CO_2_ at 37 °C. At each time point, the cells were washed with the PBS and transferred to a 15 mL centrifuge tube containing media cells and then were resuspended in 500 μL bind buffer, followed by the addition of 5 μL each of Annexin V-FITC and PI. After 15 min of incubation at room temperature, quantification of the apoptotic and necrotic population of cells was conducted by flow cytometry using an instrument from BD Biosciences, San Jose, CA, USA.

### 3.6. In Vitro Wound Healing Assay

To evaluate the migration of AMF-treated cells, a wound healing assay was performed as reported previously [36]. Briefly, 5 × 10^6^ AMF treatment-surviving HCT116 cells were plated in a 6-well plate, and after proper adherence of cells to the bottom surface, an artificial wound using a sterile 10 µL tip was created, and subsequently the debris and floating cells were removed. The scratches for four cell sets (C, F, P, and P + F) were created and 0 h images were captured using an optical microscope. The cells were provided with suitable media and maintained in a CO_2_ incubator for 24 h and the images were captured. Closure of the wound was calculated using ImageJ software Version 1.54h, and the results were presented as percentage of wound closure with respect to the untreated control.

### 3.7. Cell Colony Formation Assay

HCT116 AMF-treated cells at a density of 10^4^ were plated in a 6-well plate and were allowed to be adhered to the bottom surface of the plate. The culture medium of the cells was replaced every 48 h; after one week, colonies that developed from the single cell were stained with 0.5% crystal violet solution mixed with methanol, which helped to fix the cells. The stained colonies of cells in each well were counted using a magnifying glass or microscope to determine the percent of inhibition of the clonogenic property of malignant cells in comparison with the untreated control.

### 3.8. Statistical Analysis

The analyzed data of all the experiments in the present study are represented as mean ± standard error of three or more than three independent experiments and were analyzed by one-way ANOVA. A two-sided value equal to or less than a *p*-value < 0.01 was considered a significant value in all the in vitro experiments. All statistical calculations were performed in GraphPad Prism software version 8, and a *p*-value of < 0.01 was considered significant.

## 4. Conclusions

The PEG-coated ferrite-based nanoparticles of compositions Co_0.2_Mn_0.6_Zn_0.2_Fe_2_O_4_ (X1), Co_0.4_Mn_0.4_Zn_0.2_Fe_2_O_4_ (X2), and Co_0.6_Mn_0.2_Zn_0.2_Fe_2_O_4_ (X3) were synthesized by the one-step coprecipitation method and characterized with XRD, Raman, TEM, XPS, and VSM. Based on high saturation magnetization and SAR values, X2 nanoparticles using AMF were evaluated for antiproliferative activity against colorectal cancer cells. Our findings showed that X2 nanoparticles using AMF exhibit a significant decrease in cell viability, a decrease in colony-forming ability, abrogation of cell motility, and induction of apoptosis-mediated cell death in colorectal cancer cells. This indicates that PEG-coated ferrite nanoparticles using AMF promote a strong antiproliferative effect against colorectal cancer cells. Additionally, these results provide appealing evidence for the further evaluation of PEG-coated ferrite nanoparticles in preclinical studies as potential ferrite nanoparticles for treating colorectal and other cancers.

## Figures and Tables

**Table 1:** Refined structural parameters of the X1, X2, and X3 nanoparticles.

Refinement Parameters	X1	X2	X3
Lattice constant (a = b = c) (Å)	8.3263	8.4140	8.3865
α = β= γ (°)	90	90	90
Density (g/cm^3^)	5.705	5.528	5.583
V (Å^3^)	577.2321	595.6663	589.8453
Bragg R-factor	2.062	1.542	2.087
Rf-factor	1.808	1.268	1.758
Chi2	1.21	1.10	1.12
Average sizes (nm)	15.2	10.2	10.3

**Table 2:** The peak positions and intensity ratios A_1g_(1)/A_1g_(2) and vibrational bands of X1, X2, and X3 nanoparticles.

Composition	A_1g_(1)	A_1g_(2)	T_2g_(1)	E_g_	T_2g_(3)	I_A1g_(1)/I_A1g_(2)
X1	605.4		468.1	284.4	166.1	
X2	662.8	607.5	464.9	296.9	178.3	0.85
X3	678.6	617.3	472.3	315.9	223.6	1.07

**Table 3:** Coercive field, remnant magnetization, and saturation magnetization of X1, X2, and X3 nanoparticles at 5 and 300 K.

Composition	Coercive Field (Oe)		Remnant Magnetization (emu/g)		M_s_ (emu/g)		K_eff_ (10^3^ Jm^−3^)
	5 K	300 K	5 K	300 K	5 K	300 K	300 K
X1	4468.6	139.8	56.18	8.12	85.99	61.23	9.52
X2	4380.1	6.8	61.62	0.50	102.96	74.28	11.38
X3	6382.2	7.3	60.92	0.49	94.63	69.72	10.84

**Table 4:** List of the amounts of Co, Mn, Zn, and Fe salts used for the synthesis of X1, X2, and X3 nanoparticles.

	CoCl_2_.6H_2_O (g)	MnCl_2_ (g)	ZnCl_2_ (g)	FeCl_3_ (g)
Co_0.2_Mn_0.6_Zn_0.2_Fe_2_O_4_	0.5094	1.2712	0.2925	3.4728
Co_0.4_Mn_0.4_Zn_0.2_Fe_2_O_4_	1.0153	0.8446	0.2915	3.4610
Co_0.6_Mn_0.2_Zn_0.2_Fe_2_O_4_	1.5179	0.4208	0.2905	3.4493

## Data Availability

Data is contained within the article and Supplementary Material.

## Supplementary Materials

The following supporting information can be downloaded at https://www.mdpi.com/article/10.3390/ph17030327/s1: Figure S1: (a) hydrodynamic sizes of the nanoparticles obtained from PEG coated nanoparticles. (b,c,d) zeta potential analyses as synthesized and PEG coated nanoparticles; Figure S2: (a,b,c) FTIR spectra of as synthesized and PEG-coated X1, X2, and X3 nanoparticles; Figure S3: XPS spectra of (a) Fe^3+^, (b) Mn^2+^, (c) Co^2+^, and (d) Zn^2+^ from X1, X2, and X3 nanoparticles; Table S1: XPS peak position of divalent and trivalent cations.
