# Supplementary material for: Evaluation of Antiproliferative Properties of CoMnZn-Fe2O4 Ferrite Nanoparticles in Colorectal Cancer Cells
Source: Pharmaceuticals (Basel). 2024 Mar 1;17(3):327. doi: 10.3390/ph17030327 (PMC10973991; doi:10.3390/ph17030327)
Supplement: Supplementary file 1 [file pharmaceuticals-17-00327-s001.zip › pharmaceuticals-2871119-supplementary.pdf]

Supplementary data

- *DLS and zeta potential analyses of nanoparticles*

Due to the high specific surface area and magnetic interaction between nanoparticles, they could easily be aggregated. Therefore, coating nanoparticles with biocompatible polymer PEG (i.e., PEGylation process) is an effective way to improve the colloidal stability of dispersions containing ferrite nanoparticles in biological media and a magnetic field. The hydrodynamic sizes of the X1, X2, and X3 nanoparticles are shown in Figure S1(a), the sizes vary in the range of 100-200 nm. The zeta potential of the synthesized and PEG coated nanoparticles are shown in Figure S1(b, c, d). The zeta potential of the PEG coated nanoparticles shifted to lower values −40 to −60 mV and the synthesized nanoparticles have a zeta potential value of −30 to −38 mV.

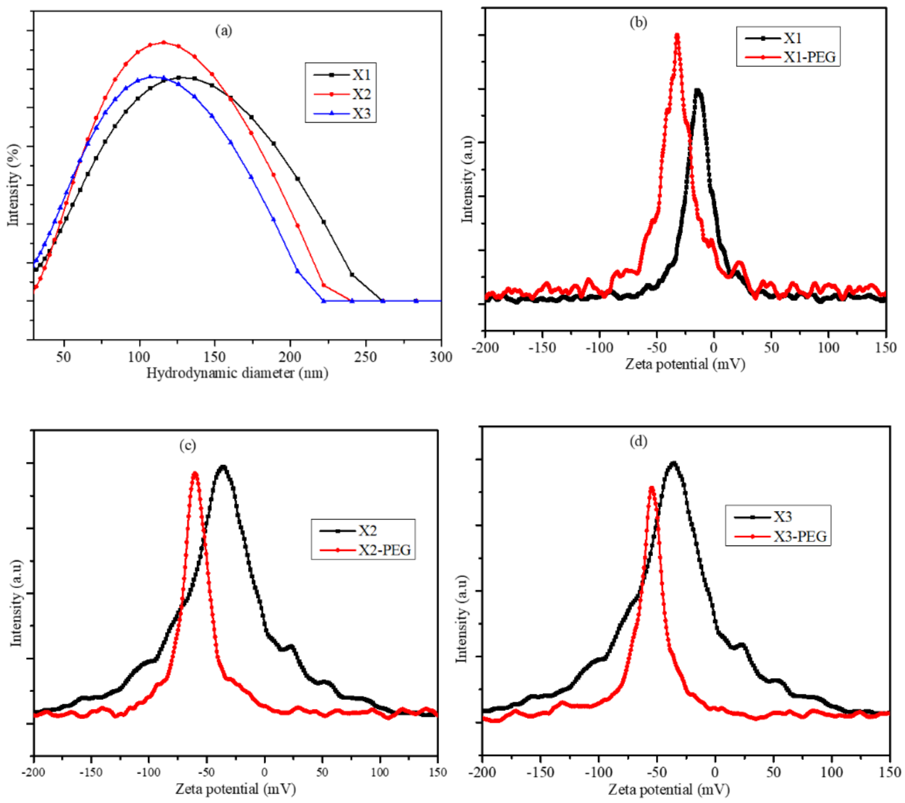

Figure S1 (a) hydrodynamic sizes of the nanoparticles obtained from PEG coated nanoparticles. (b,c,d) zeta potential analyses as synthesized and PEG coated nanoparticles.

- *FTIR analysis of as synthesized and PEG coated X1, X2, and X3 nanoparticles.*

FTIR analysis was used to identify PEG in functionalized ferrite nanoparticles. The FTIR spectra of synthesized nanoparticles and PEG coated nanoparticles are shown in Figure S2(a,b, c). In the FTIR spectra of PEG coated ferrite nanoparticles additional peaks corresponding to bending vibration of -C-O-C- (1100 -1250 cm^{-1}), out-of-plane bending vibration of -CH (833 cm^{-1}) and bending vibrations of $-CH_2$ (1385 cm^{-1}) are observed.

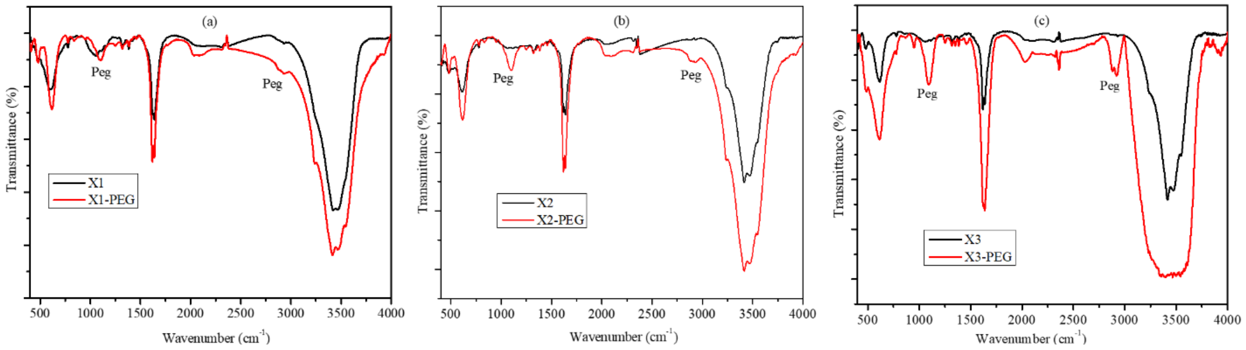

Figure S2. (a,b,c) FTIR spectra of as synthesized and PEG-coated X1, X2, and X3 nanoparticles.

- *XPS of X1, X2, and X3 nanoparticles*

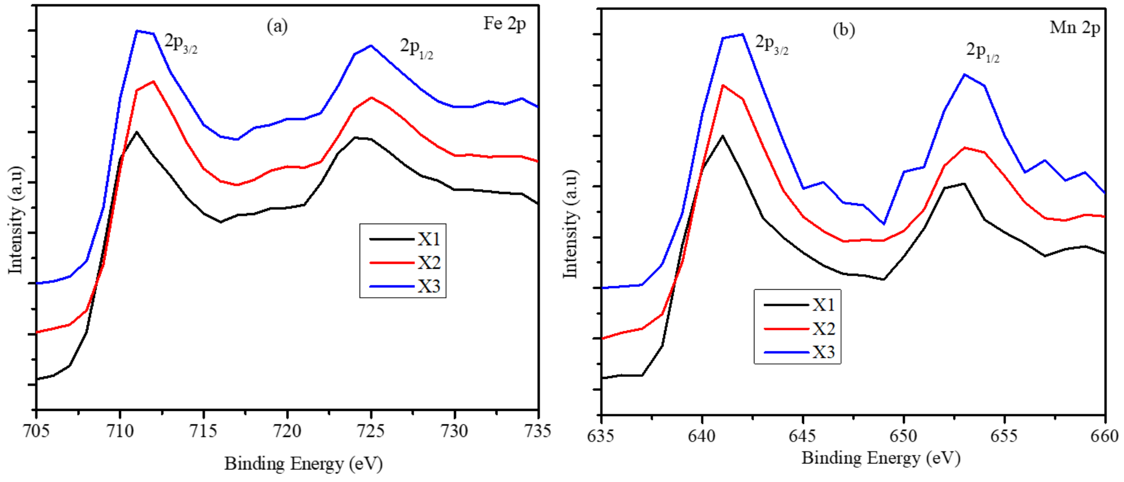

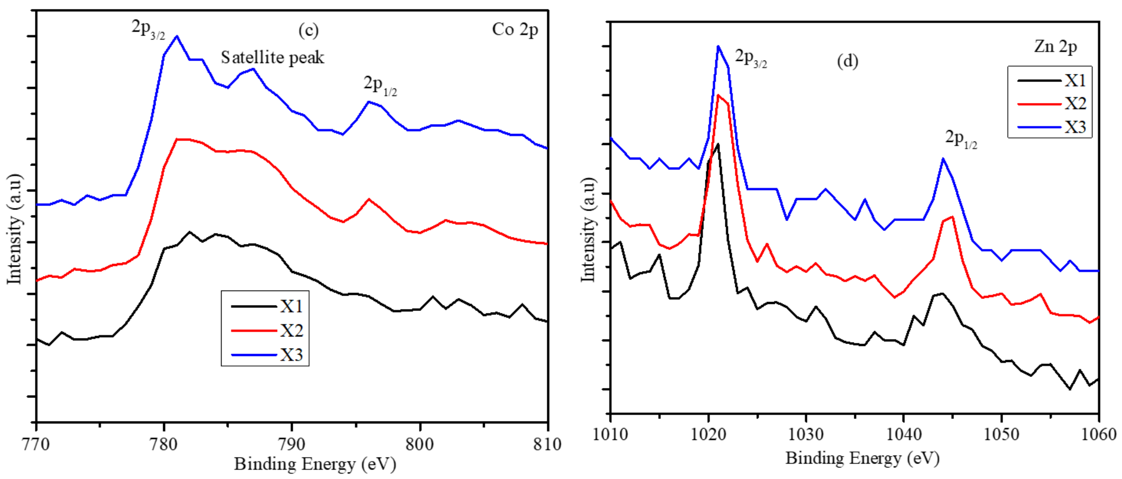

Figure S3. XPS spectra of (a) Fe^{3+}, (b) Mn^{2+}, (c) Co^{2+}, and (d) Zn^{2+} from X1, X2, and X3 nanoparticles.

The XPS spectra of the Fe^{3+}, Mn^{2+}, Co^{2+}, and Zn^{2+} cations are shown in Figure S3. XPS spectra obtained for both the trivalent and divalent metal cations from X1, X2, and X3 nanoparticles. The peak positions of the individual cations in three sets of nanoparticles are comparable with slight composition dependent shift which is attributed to the valence state and cations present in the neighboring lattice positions. The characteristic peak positions and identical peak patterns in the XPS spectra showed uniform distribution of divalent cations in the spinel lattice and the absence of any additional phases of ZnO, Co_3O_4, and MnO_2. The positions of the $2p_{3/2}$, and $2p_{1/2}$ peaks corresponding to the cations are listed in Table S1. The Fe^{3+} showed a major peak at 711.0 eV attributed to the peak from $Fe2p_{3/2}$ core level electrons of the Fe^{3+}. The intensities of the peaks indicate the mass percentage of Fe^{3+} is identical in the three set of nanoparticles. The peaks corresponding to the Mn^{2+} and Co^{2+} shown in Figure S3(b, c) show concentration dependent intensity particularly the satellite peak Co^{2+} is more significant in the spectra of X3 nanoparticles due to the higher mass percentage of Co. The Zn^{2+} peaks have the identical intensity and slight concentration dependent shift in the peaks in which $2p_{3/2}$ and $2p_{1/2}$ peaks shift to higher binding energy with the increase in the Co^{2+} atomic percentage.

XPS Peaks	Fe^{3+} (eV)	Co^{2+} (eV)	Mn^{2+} (eV)	Zn^{2+} (eV)
$2p_{3/2}$	711.0	781.0	641.5	1020.0
$2p_{1/2}$	725.0	796.0	653.0	1044.3

Table S1. XPS peak position of divalent and trivalent cations.
